# Rare presentation and resection of giant cell tumor invading the middle ear and skull base: A case report

**DOI:** 10.1016/j.jpra.2025.01.020

**Published:** 2025-02-01

**Authors:** Joshua M. Cohen, Geena Jung, Hailey Reisert, Yi-Hsueh Lu, Genesis Liriano, Bradley A. Schiff, Evan S. Garfein, Andrew J. Kobets

**Affiliations:** a Montefiore Medical Center, Albert Einstein College of Medicine, Bronx, NY, USA; b Department of Plastic and Reconstructive Surgery, Montefiore Medical Center, Bronx, NY, USA; c Department of Neurosurgery, Montefiore Medical Center, Bronx, NY, USA; d Department of Otolaryngology, Montefiore Medical Center, Bronx, NY, USA

**Keywords:** Giant cell tumor, Giant cell granuloma, Skull base, Middle ear, Cervicofacial flap, Multidisciplinary

## Abstract

Giant cell tumors (GCTs) are common benign bone tumors found in young adults, frequently occurring in the long bones but rarely in the skull. Although considered benign, GCTs have the potential to be aggressive, destroying surrounding tissue. Here, we present the unusual case of a patient with a GCT of the middle ear and skull base. A 19-year-old male with an extensive giant cell tumor underwent surgical resection by a multidisciplinary team consisting of otolaryngology, neurosurgery, and plastic and reconstructive surgery. The mass was grossly resected, and a cervicofacial flap allowed for reconstruction with optimal cosmesis, a procedure not described for GCTs. This approach allowed for excellent wound healing without tumor recurrence. Gross total resection remains the most effective treatment for GCTs, minimizing radiation exposure and recurrence risk. However, an optimal surgical approach for skull GCTs has yet to be described. Skull-base GCTs present a challenge due to their complex anatomy and neurovascular elements. Further, the high rates of local recurrence make gross resection the goal of treatment, though this is difficult to achieve. The present case was successfully managed by extensive multidisciplinary surgical resection, highlighting the necessity of collaborative effects to achieve gross total resection and positive aesthetic outcomes.

## Introduction

Giant cell tumors (GCT) are rare, benign lesions derived from undifferentiated mesenchymal cells of the bone marrow. These tumors account for about 5% of primary and 20% of benign bone tumors, typically arising in the long bone epiphyses.^1^ Only 2% of GCTs occur in the head and neck region, with less than 1% in the skull, most commonly in the sphenoid or temporal bones.^1^, ^2^, ^3^ Presenting symptoms vary with location and may include otalgia, hearing loss, tinnitus, localized swelling, and facial paralysis**.**^3^

Despite being benign, GCTs are aggressive, with recurrence rates of 40%−60%.^4^

Surgical treatment of skull base GCTs is often associated with considerable morbidity, prompting the exploration of various surgical and non-surgical approaches.^5^^,^^6^ Gross total resection (GTR) has shown curative potential, though difficulty varies widely due to GCTs' aggressive nature.

We present a novel three-discipline approach for resecting an extensive GCT of the middle ear and skull base, involving neurosurgery, otolaryngology, and plastic and reconstructive surgery in a single procedure. The unique presentation, successful resection, and excellent aesthetic outcome highlight this method's potential in future cases.

## Case presentation

### Initial presentation

**Figure 1:**
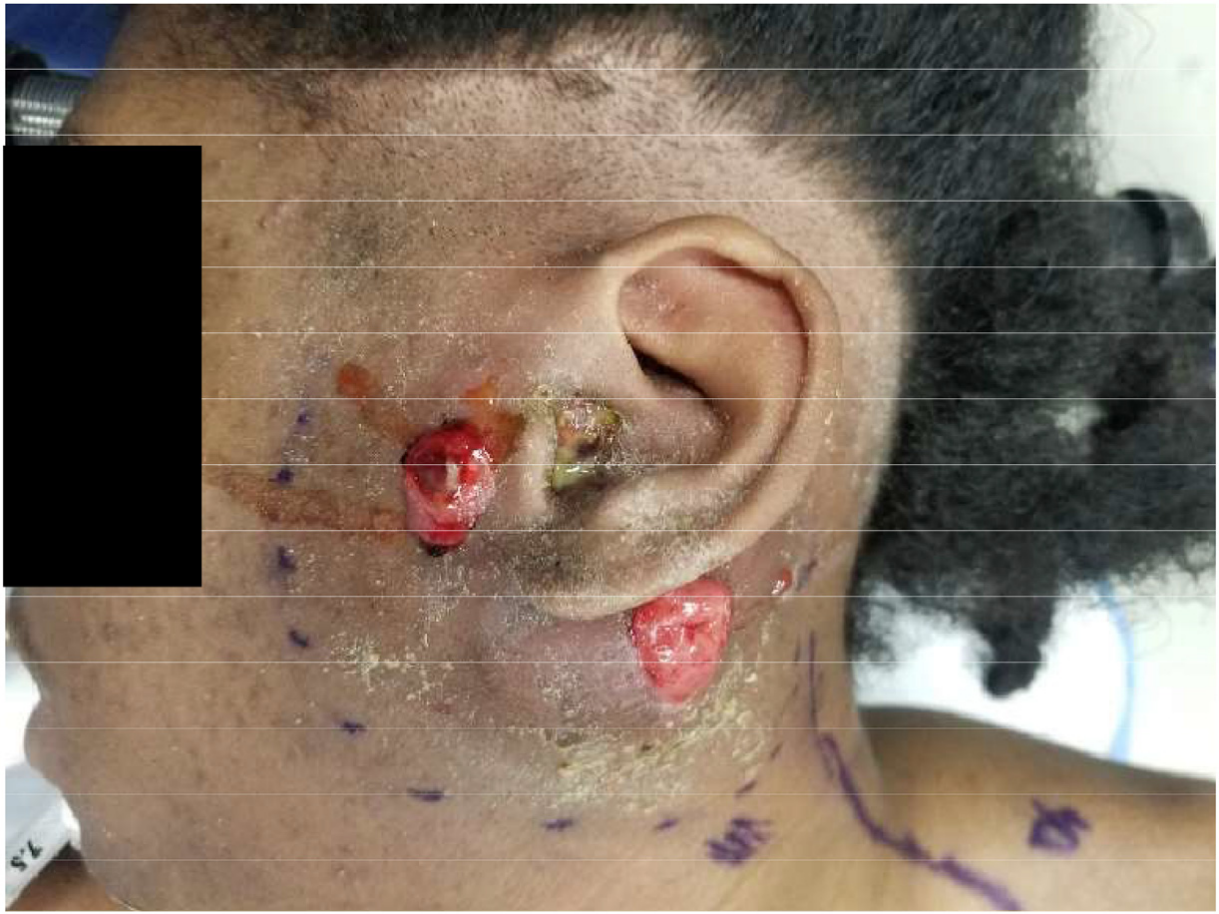
Pre-operative image demonstrating the mass protruding anteriorly and posteriorly to the left outer ear.

A 19-year-old male presented with left-sided facial paralysis and otorrhea following a 3-month history of ear pain and fullness. Three months prior, he was treated at an urgent care with antibiotic ear drops for a presumed ear infection, which temporarily resolved his symptoms. At this point, no imaging was conducted as the condition was thought to be a simple ear infection. Two months later, he sustained a hit to the left side of his head while playing basketball, leading to significant pain and subsequent left-sided facial paralysis, along with a recurrence of his ear pain and fullness. He then presented to the local Emergency Department, where he was started on vancomycin and cefepime. On admission, a mass was visibly protruding near the left ear (Figure 1).

**Figure 2:**
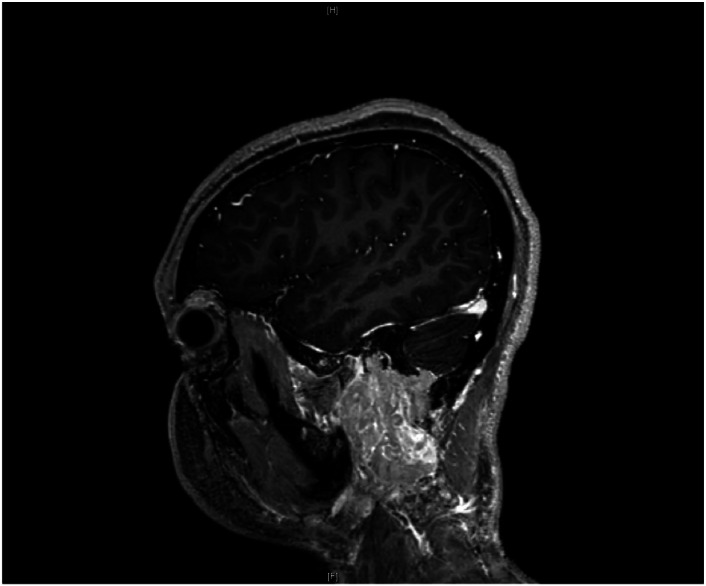
Sagittal MRI demonstrating the large invasive Giant Cell Tumor (GCT). The mass is noted to be extending through the middle ear, into the skull base, and displaces the parotid gland.

Imaging with MRI and CT revealed a large soft tissue mass (5 × 4 × 3 cm) with central necrosis and hemorrhage centered around the left mastoid, compressing the sigmoid sinus and displacing the parotid gland. MRI of the internal auditory canal (IAC) showed the mass abutting the course of the facial nerve and cerebellum adjacent to the sigmoid sinus (Figure 2). Magnetic resonance venography (MRV) results were concerning for glomus valage and attenuation of the left internal jugular vein due to mass effect and hypoplasia of the left transverse sinus. Soft tissue biopsy confirmed multinucleated giant cells.

### Surgical management

**Figure 3:**
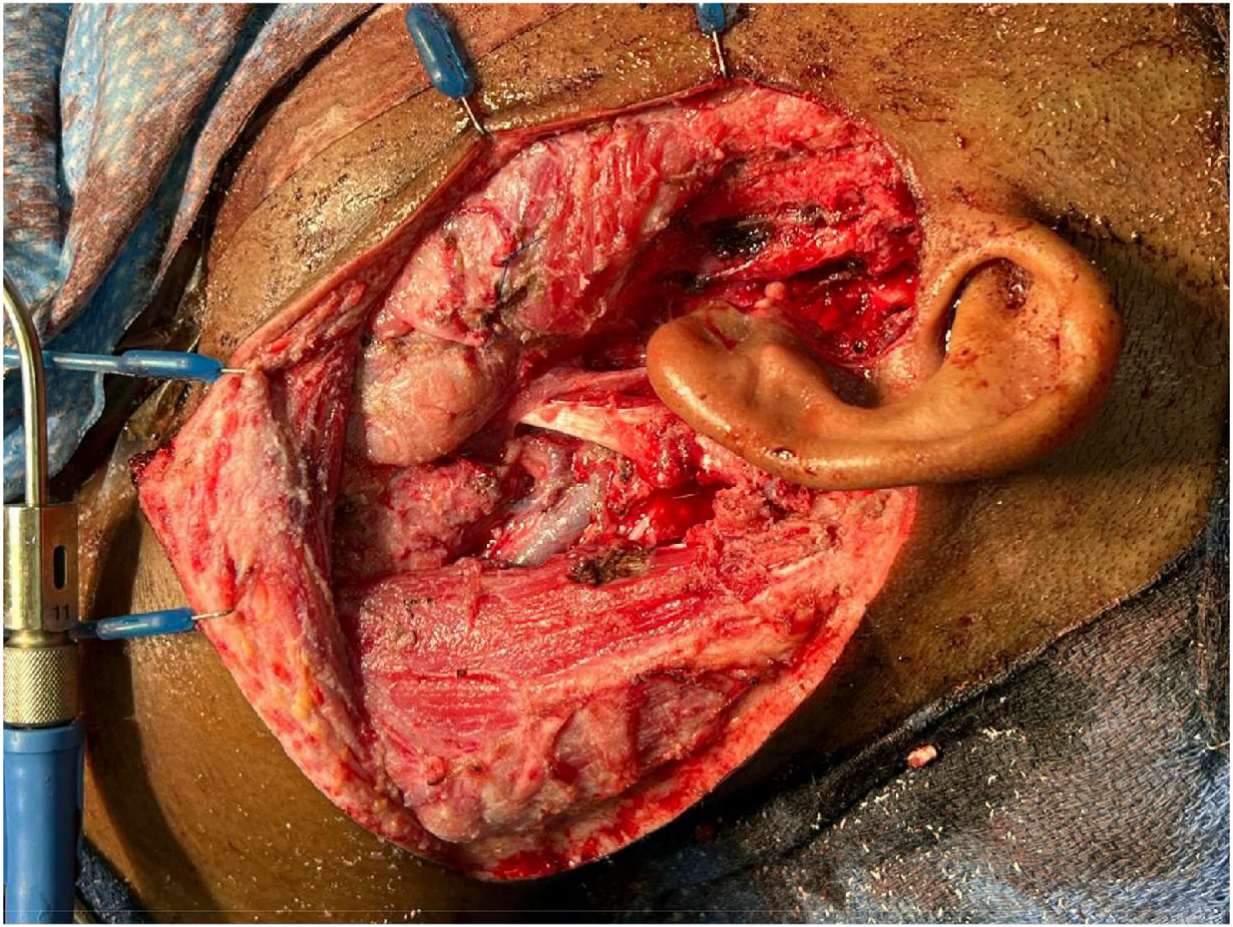
Intraoperative image demonstrating the depth of tumor invasion and adhesion to the facial nerve, parotid gland, and temporal bone.

A multidisciplinary approach involving otolaryngology, neurosurgery, and plastic and reconstructive surgery was planned. Otolaryngology began the procedure with an extensive neck dissection, removing the anterior portion of the tumor. A curvilinear periauricular extended to the mid-chest to incorporate the site of the donor rotational flap (Figure 3). The left platysma was identified and divided, subplatysmal flaps raised, and the greater auricular nerve resected.

The surgical team then proceeded with the total parotidectomy. The mass was seen to be invading all divisions and branches of the facial nerve, which, therefore, had to be sacrificed. Facial nerve repair with a graft was not possible due to the proximal involvement of the nerve. Lymph nodes from level 2 to the internal jugular vein were removed. The tumor was freed from gross negative margins except for the lesion left on the sigmoid sinus.

The neurosurgery team resected the tumor from the skull base by drilling the occipital bone. It was adherent to the intracranial sinus, and complete removal of the mass risked entry into the sinus and massive hemorrhage. To limit complication risks, adherent tumor was cauterized with a low bipolar setting. After removal, the sinus refilled from its prior compressed state. A small mass focus adherent to the dura over the sinus was coagulated and lifted to avoid invasion or intracranial extension. In similar cases, bone invasion can be missed in the craniectomy, and thus, good margins were obtained in the bone when not functionally debilitating. Due to the small invasive portion of the mass, skull base reconstruction was not necessary, and replacement was not indicated. Finally, the wound was irrigated with antibiotic saline.

**Figure 4:**
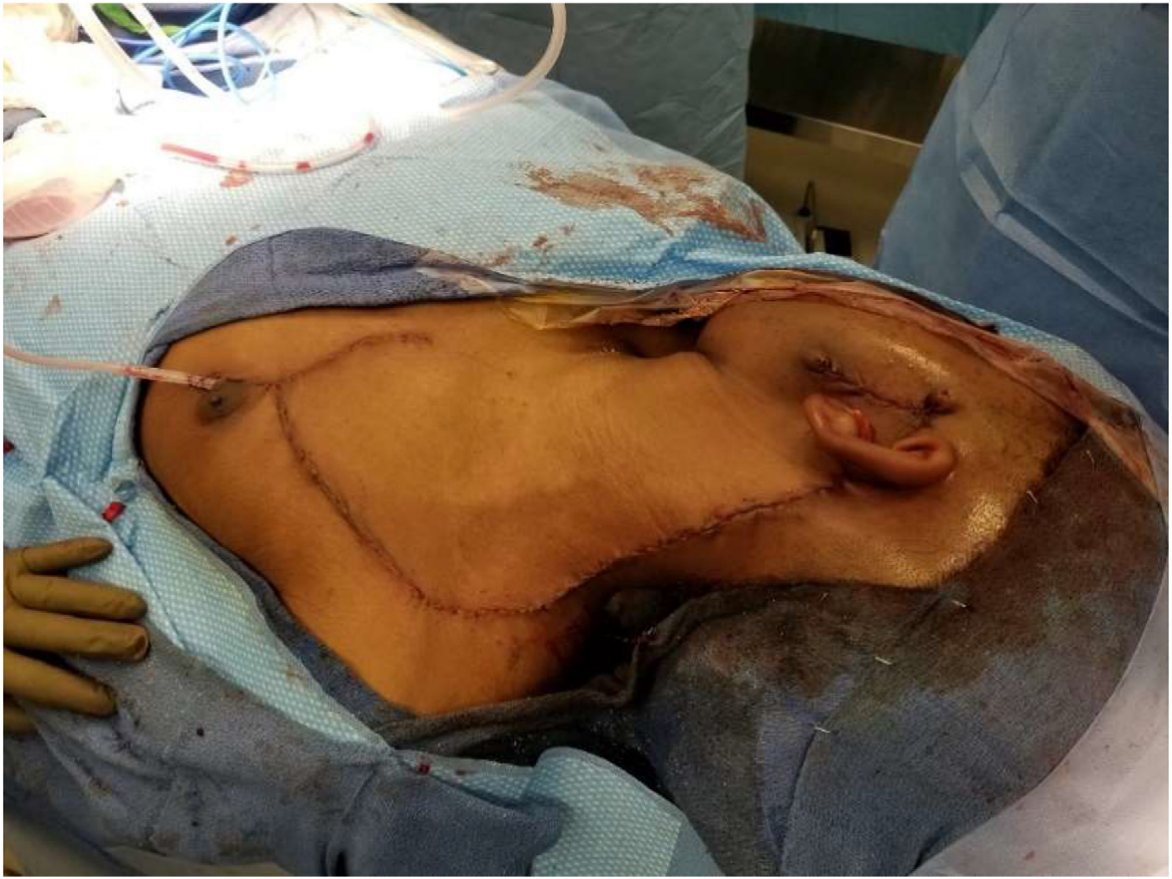
Post-operative image demonstrating the cervicofacial flap with aesthetic deformity correction.

Following resection of the tumor, the defect involved the skin and soft tissue of the left cheek and neck with exposed skull base. A large cervicofacial flap was elevated, supplied by the transverse cervical artery and the second intercostal artery perforator. A backcut was performed to allow tension-free rotation of the flap into the defect. Using a local fasciocutaneous flap yielded an excellent skin color and quality match, with minimal donor site morbidity (Figure 4).

### Postoperative course

**Figure 5:**
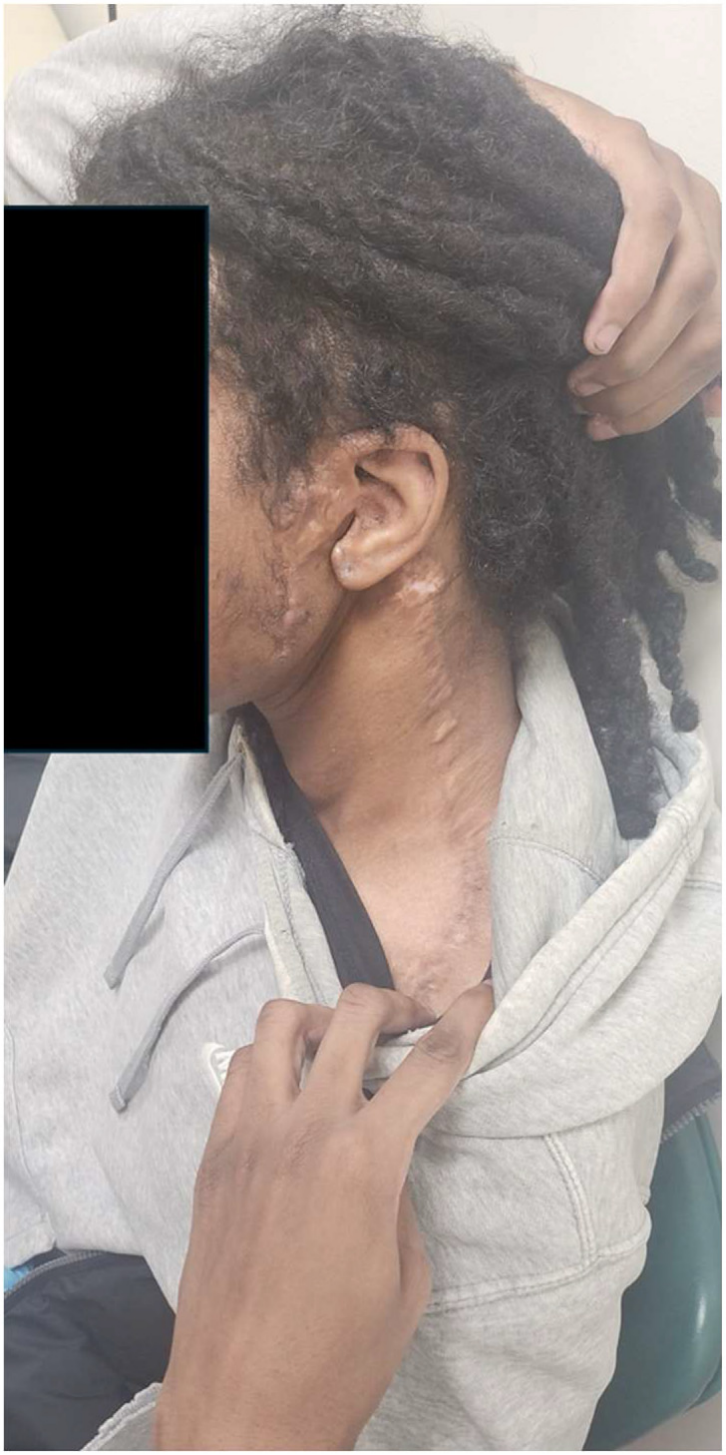
Image taken at 38 months post-operatively to demonstrate continued excellent healing.

No acute postoperative complications occurred, and the patient was discharged. Follow-up revealed excellent wound healing with no postoperative pain or infection. He continued to experience moderate left-sided hearing loss, attributed to middle ear invasion. Left CN VII palsy resulted in lagophthalmos, treated two months later with a platinum eyelid weight and lid adhesion. Thirty-two months after the initial procedure, the patient presented with a desire to fix his remaining facial asymmetry, undergoing a static sling using Alloderm, which provided satisfactory aesthetic results. Further dynamic reconstruction, which would have required staged procedures, not pursued. Follow-up imaging over three years showed no recurrence of the mass. His incisions healed with an excellent aesthetic result (Figure 5).

## Discussion

The present case describes a novel three-discipline technique to repair a massively extensive tumor with excellent resection and optimal cosmesis. The procedure required an extensive and nuanced multi-disciplinary approach that permitted safe resection from around the mastoid cavity, skull base, and upper neck. The novel utilization of the cervical skin flap led to an eloquent and aesthetic reconstruction of the lateral face and neck without complication. The patient's post-resection recovery included elective procedures to address facial nerve paralysis and facial symmetry, both of which resulted in successful functional and cosmetic outcomes.

Surgical resection remains the primary treatment for GCTs due to the tumor's aggressive nature and high recurrence risk, as non-surgical options, including embolization, radiation, and denosumab, have shown limited efficacy.^6^, ^7^, ^8^ The extensive invasion of our case's tumor necessitated our multidisciplinary approach, combining neurosurgical and otolaryngological teams for the initial resection to enhance the safety and efficacy of the procedure. Further, the involvement of plastic and reconstructive surgery aimed to fulfill the need for skin coverage while maximizing functional preservation. The operative approach for GCT resection in cases similarly expansive to the one described here should be carefully selected under the guidance of an integrated perspective.

While skull base GCT resection poses risks, including hemorrhage, cranial nerve deficits, and cosmetic concerns, a tailored approach can maximize resection and minimize complications, including recurrence.^4^^,^^9^ The expansive and unique location of our case's tumor required that we utilize this distinct approach. The excellent aesthetic result concurrent with a curative resection achieved for our patient may facilitate other clinicians to utilize this methodology to manage their patients in the future.

## Conclusion

We report the successful management of an aggressive giant cell tumor using a combined approach. Attention must be paid to the sensitive vascular and neurological structures in the extralesional tissue. An autologous cervical skin flap can provide a durable and aesthetically pleasing reconstruction of the pre- and post-auricular areas and the cervical incision sites.

## IRB approval

Institutional Review Board permission were obtained for case publication.

## Ethical approval

Not required.

## Funding

None.

## Patient consent

Written consent has been obtained for publication, with identifying features removed from the figures and manuscript.

## Declaration of competing interest

This research did not receive any specific grant from funding agencies in the public, commercial, or not-for-profit sectors.
